# Green Extraction of Pectin from Sugar Beet Flakes and Its Application in Hydrogels and Cryogels

**DOI:** 10.3390/gels10040228

**Published:** 2024-03-27

**Authors:** Florina Dranca, Silvia Mironeasa

**Affiliations:** Faculty of Food Engineering, Stefan cel Mare University of Suceava, 720229 Suceava, Romania; silviam@fia.usv.ro

**Keywords:** sugar beet flakes, pectin, extraction, physicochemical properties, hydrogel, cryogel

## Abstract

Sugar beet flakes, a by-product of the sugar industry, were used as a source for pectin extraction that was performed using conventional citric acid extraction (CE) and two non-conventional extraction techniques—microwave-assisted extraction (MAE) and pulsed ultrasound-assisted extraction (PUAE). The influence of extraction conditions was studied for each technique based on pectin yield and galacturonic acid content, and spectroscopic, chromatographic and colorimetric methods were used for pectin characterization. Better results for pectin yield were achieved through CE (20.80%), while higher galacturonic acid content was measured in pectin extracted using PUAE (88.53 g/100 g). Pectin extracted using PUAE also presented a higher degree of methylation and acetylation. A significant increase in the molecular weight of pectin was observed for the PUAE process (7.40 × 10^5^ g/mol) by comparison with conventional extraction (1.18 × 10^5^ g/mol). Hydrogels and cryogels prepared with pectin from sugar beet flakes also showed differences in physicochemical parameters determined by the method of pectin extraction. Hydrogels had higher bulk density values irrespective of the pectin extraction method, and overall lower values of the textural parameters. Cryogels prepared with pectin from CE showed higher values of the textural parameters of hardness, adhesiveness, cohesiveness, gumminess and chewiness, while gels obtained with pectin from MAE and PUAE had higher thermal stability. The results of this study prove that sugar beet flakes can be considered a potential source for pectin production, and the extracted pectin is suitable for obtaining hydrogels and cryogels with physicochemical parameters comparable to the commercial citrus and apple pectin available on the market.

## 1. Introduction

Pectin is a naturally occurring polysaccharide and is a main component of the primary plant cell wall and middle lamella of higher plants [1,2]. Due to its unique properties, pectin extracted from plant materials and purified has various applications in the production of food, cosmetics and pharmaceutical products [3]. In the food industry, pectin has been approved by regulatory authorities as a food additive, is included on the list of “Direct food substances affirmed as generally recognized as safe” by the United States Food and Drug Administration, and is classified within the European Union as food additive E440 by the European Food Safety Authority. Pectin is used as a gelling, thickening, stabilizing and emulsifying agent in products such as beverages, fruit-based products, fillings for bakery and confectionary products, yogurt and meat products [4,5].

Commercial pectin is mainly extracted from citrus peel and apple pomace, which makes its industrial production dependent on the availability and cost of these raw materials. The current dependency of the pectin market on citrus peel and apple pomace, together with the increasing demand for pectin and the diversification of its applications (encapsulating agent [6], 3D printed food component [7], metal binding [8], development of tissue-engineered scaffolds [9]) has prompted research into new raw materials that can be used as pectin sources. In a study of pectin content and composition in 26 different food waste streams from industrial processing plants in Europe, sugar beet flakes were considered a source of pectin with high degree of methylation and acetylation [10]. Sugar beet flakes or sugar beet shreds are by-products of sugar beet processing in the sugar industry and are mainly composed of polysaccharides (22–24% cellulose, 30% hemicelluloses and 15–25% pectin on dry basis), and small amounts of protein, fat, ash and lignin (approximately 21% on dry basis) [11]. In general, 1 tonne of sugar beets processed in the sugar industry yields 130–160 kg of refined sugar and 50 kg of dehydrated sugar beet flakes. Considering that the European Union reported a production of 14.2 million tonnes of beet sugar during the 2020/21 campaign [12], it can be estimated that around 4 million tonnes of sugar beet flakes were generated by the production process. Sugar beet flakes are mostly used as animal feed, despite the high pectin content and availability in large quantities, which make this by-product a suitable pectin source.

Pectin production is a process that comprises at least three main stages: plant pretreatment, extraction and purification. The extraction process usually employs the use of mineral acids and a heat treatment. Because of the challenges associated with this type of extraction process, which include the use of large quantities of mineral acid that poses environmental risks, the length of the process, low extraction selectivity and the risk of degradation of heat-labile compounds, non-conventional green extraction techniques have been proposed and applied to extract pectin from different plant materials [13]. Ultrasound-assisted extraction, microwave-assisted extraction and subcritical water extraction are green techniques that are being explored for their ability to extract pectin from plant materials without posing great environmental risks [14].

Recent advances in the study of pectin have also involved the diversification of its applications. In the last years, the gelling properties of pectin have gained increased interest as numerous studies have investigated the use of this polysaccharide for the preparation of hydrogels. Hydrogels are defined as three-dimensional hydrophilic polymer networks capable of absorbing and retaining water [15]. According to the method of preparation and the drying process, specific gel types can be obtained using pectin. Starting from the hydrogel structure, different drying procedures result in different porous materials such as cryogels (hydrogels dried by lyophilization or supercritical drying), aerogels (hydrogels dried by substituting the liquid with air) and xerogels (oven- or air-dried hydrogels). Furthermore, when a pectin solution is mixed with oil and the resulting emulsion is freeze-dried, oleogels can be obtained. Most of the applications of pectin hydrogels to date refer to their use as drug delivery systems [16]; however, recent studies have also reported on their applications in the food industry [17,18,19]. It is important to emphasize that pectin hydrogels can enhance the structural or textural properties of foods and can mimic the desired texture of food products, and therefore are of great interest in modern food technology. Recent studies have also examined the use of pectin in hydrogels that serve as food-ink formulations for 3-D printing [7,20].

This study aims to explore conventional (CE) and non-conventional extraction techniques (microwave-assisted extraction (MAE) and pulsed ultrasound-assisted extraction (PUAE)) applied to obtain pectin from sugar beet flakes, to compare the physicochemical properties of the extracted sugar beet pectin to the properties of commercial pectin, and finally to investigate the applications of pectin from sugar beet flakes for hydrogel and cryogel preparation.

## 2. Results and Discussion

### 2.1. The Influence of Extraction Conditions on Pectin Yield and Galacturonic Acid Content

The model used to predict the evolution of pectin yield and galacturonic acid (GalA) content for pectin extraction by CE, MAE and PUAE was a second-order (quadratic) polynomial response surface model that was selected because of the higher coefficient of determination (*R^2^*), both adjusted (*Adj-R^2^*) and predicted (*Pred-R^2^*), and the low value of *p (*<0.0001). The ANOVA results (Tables S4–S6, Supplementary Materials) indicate a satisfactory representation of data by the models at a 95% confidence level; the quadratic model can explain and predict most of the variation of pectin yield (*R^2^* = 0.9849 for CE, *R^2^* = 0.9898 for MAE, and *R^2^* = 0.9843 for PUAE) and galacturonic acid content (*R^2^* = 0.9642 for CE, *R^2^* = 0.9867 for MAE, and *R^2^* = 0.9922 for PUAE).

**Figure 1:**
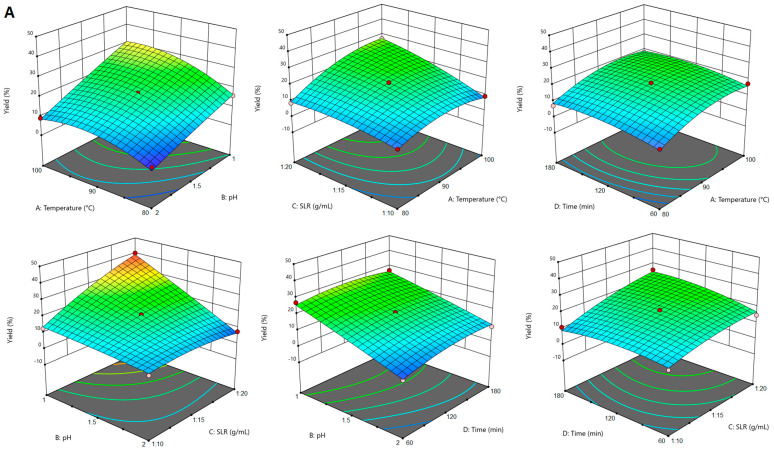

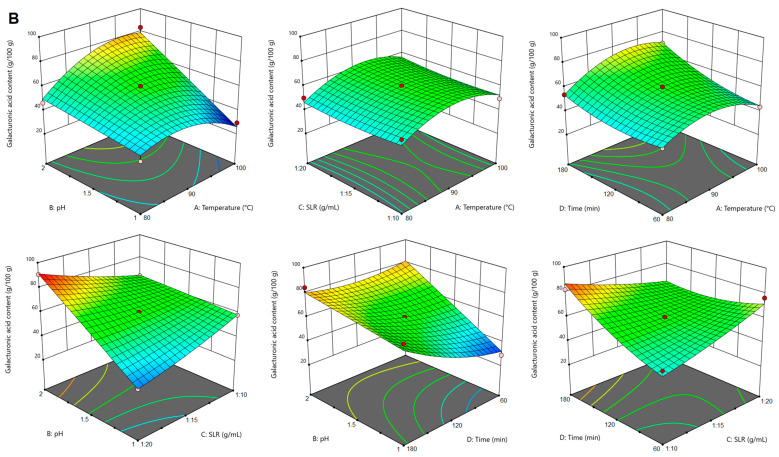
Response surface plots showing the effects of CE parameters on pectin yield (**A**) and galacturonic acid content (**B**).

For conventional citric acid extraction of pectin from sugar beet flakes, it was observed that the yield was significantly influenced (*p* < 0.05) by all the extraction parameters (temperature, pH, SLR and time) and by the following interactions: temperature–pH, temperature–SLR, pH–SLR and SLR–time. As shown in the 3D graphs in Figure 1A, the increase of the extraction temperature determined a higher extraction yield. This was also observed for pectin extraction from other plant materials and was attributed to higher diffusion of solvent into the solid matrix, which increases the mass transfer of pectin into the solvent [21,22]. The same tendency was observed for SLR, while the increase of pH was not correlated with a higher extraction yield. This positive evolution of the pectin yield as determined by the decrease of the extraction solvent pH has also been reported in other studies [23,24], and was correlated with the hydrolysis of insoluble protopectin into soluble pectin. Extraction time had a positive effect on pectin yield up to approximately 120 min; however, with the increase of time beyond this point, the yield was slightly reduced. It may be in this case that during prolonged extraction the solvent causes to some extent cleavage of the glycoside and ester bonds of pectin [24].

For the galacturonic acid content of pectin extracted by CE, ANOVA results show that this parameter was significantly (*p* < 0.05) influenced by temperature, pH and time, and by the temperature–pH, pH–SLR, pH–time and SLR–time interactions. Overall, the increase of temperature, pH, SLR and extraction time led to higher GalA content in the extracted pectin (Figure 1B). The effect of pH on GalA content was opposite to that observed for pectin yield, implying that a pH of 2 was advantageous in increasing the GalA content of pectin, which has also been observed for pectin from pomegranate peels [25].

**Figure 2:**
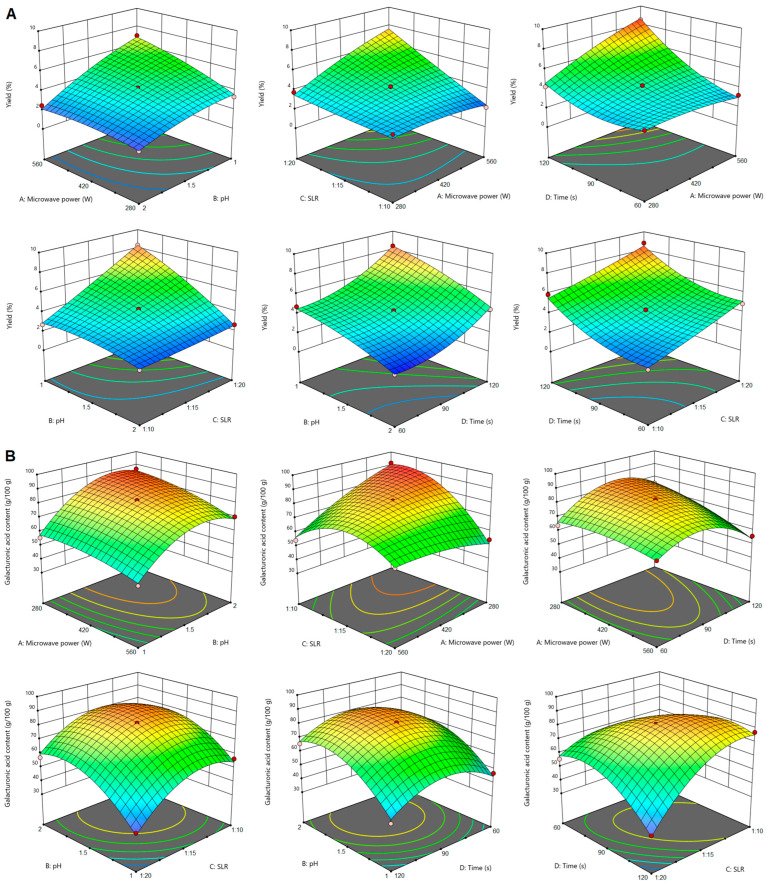
Response surface plots showing the effects of MAE parameters on pectin yield (**A**) and galacturonic acid content (**B**).

Microwave-assisted extraction is one of the two non-conventional extraction techniques that were applied to obtain pectin from sugar beet flakes. For MAE, it was observed that pectin yield was significantly (*p* < 0.05) influenced by all the extraction parameters (microwave power, pH, SLR and time) and by the power–pH, power–SLR, power–time and pH–SLR interactions. The increase of microwave power, SLR and extraction time, alongside a decrease of pH, led to an increase in pectin yield (Figure 2A). Exposure to microwaves promotes plant cell wall loosening, therefore enhancing solvent penetration into the plant matrix. Combined with a higher SLR and consequently greater surface contact between plant material and solvent, these extraction conditions increased pectin transfer into the solvent. By increasing the exposure time of the extraction mixture to microwave irradiation, the release of pectin was enhanced, as it was also observed in other studies [26,27]. An opposite effect of microwave power, pH and SLR increase was found for the galacturonic acid content of pectin. The graphs in Figure 2B show that GalA content was higher when the microwave power and SLR were at the lowest levels (280 W and respectively 1:10 g/mL) and the pH of the extraction solution was at the highest level (pH = 2). The extraction time did not influence the GalA content of pectin from sugar beet flakes, which was in line with the findings of previous studies [27]. The GalA content of pectin from sugar beet flakes was significantly (*p* < 0.05) influenced by the microwave power–SLR, power–time and SLR–time interactions.

**Figure 3:**
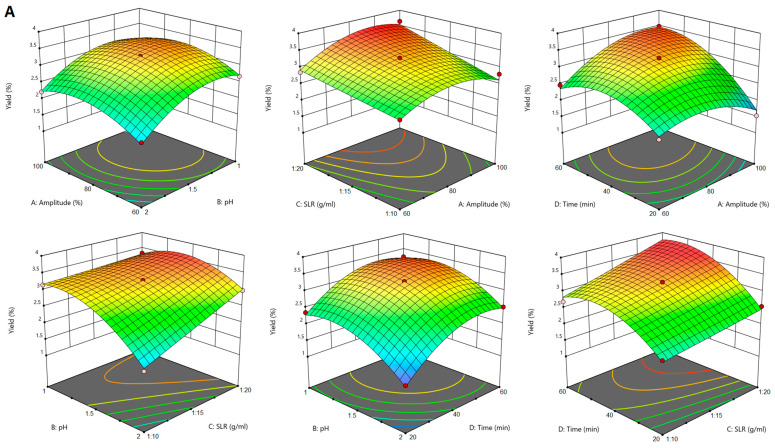

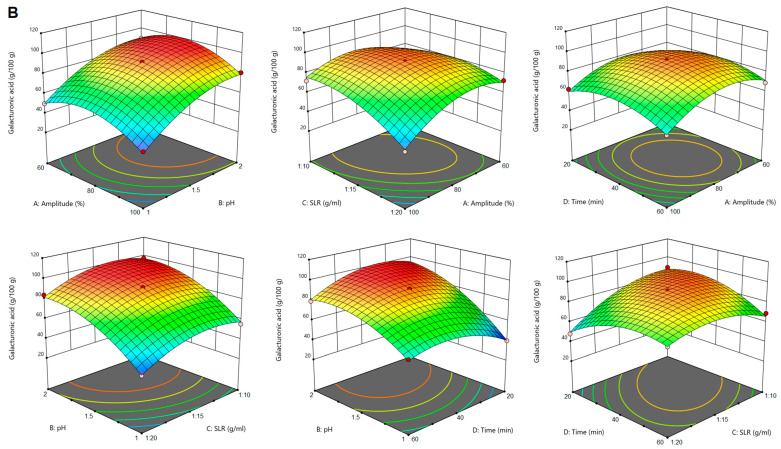
Response surface plots showing the effect of PUAE parameters on pectin yield (**A**) and galacturonic acid content (**B**).

The yield of pectin extracted by PUAE was influenced significantly (*p* < 0.05) by all the extraction parameters (ultrasound amplitude, pH, SLR and time) and by the amplitude–SLR, amplitude–time, pH–SLR and SLR–time interactions. Figure 3A shows that pectin yield increased with the increase of ultrasound amplitude, SLR and sonication time. The positive influence of ultrasound amplitude may be attributed to the larger size of active bubbles during cavitation at higher amplitudes, which provides more energy to the reaction [28,29]. The positive effect of SLR is concurrent to that of ultrasound amplitude in the sense that a larger quantity of extraction solvent relative to plant material favours the formation of more cavitation bubbles during sonication and increases the exposure of sugar beet flakes to the extraction solvent. These findings were in line with previous reports [30]. Similar to pectin extraction from sugar beet flakes by CE and MAE, in the case of PUAE pectin yield was also higher when the extraction solution had a low pH.

The GalA content of pectin from PUAE was influenced significantly (*p* < 0.05) by ultrasound amplitude, pH and SLR, and by the amplitude–SLR, pH–time and SLR–time interactions. The GalA content decreased as the amplitude increased above 60% and SLR increased above 1:15 g/mL, but it was positively influenced by an increase in pH to a value of 2. This evolution of the GalA content of pectin from PUAE as influenced by the extraction conditions resembled that of MAE pectin but was opposite to that reported for the GalA content of pectin extracted by PUAE from other sources [31].

### 2.2. Optimization and Validation of Extraction Conditions

The conditions of the conventional and non-conventional extraction methods were optimized with the purpose of reaching simultaneously a maximum pectin yield and GalA content in the extracted pectin. For the conventional citric acid extraction method, the optimal extraction conditions were a temperature of 96 °C, pH of 1.70, SLR of 1:10 g/mL and time of 180 min, which were estimated to result in an extraction yield of 21.65% and a GalA content of 76.71 g/100 g. The extraction conditions of microwave-assisted extraction were also optimized in order to maximize the yield and GalA content of pectin. The model predicted a pectin yield of 7.30% and GalA content of 66.18 g/100 g for an extraction at a microwave power of 455 W, pH of 1.45, SLR of 1:14 g/mL and extraction time of 120 s. For pulsed ultrasound-assisted extraction, the optimal extraction conditions predicted by the model to achieve a yield of 3.42% and GalA content of 91.20 g/100 g were ultrasound power of 80%, pH of 1.60, SLR of 1:16 g/mL and extraction time of 47 min.

Each extraction was repeated in triplicate in the optimal conditions presented above to validate the predictions of the Box-Behnken design. The following average values (± standard deviation) were obtained for pectin yield and GalA content: for CE, 20.80 ± 1.46% and 76.12 ± 3.18 g/100 g; for MAE, 7.24 ± 0.70% and 66.80 ± 2.02 g/100 g; and for PUAE, 3.40 ± 0.63% and 90.34 ± 3.21 g/100 g. Because these values were well correlated with the predicted values, it was concluded that the optimal conditions for CE, MAE and PUAE of pectin from sugar beet flakes were valid.

### 2.3. Comparison of Extraction Methods Based on Yield and Galacturonic Acid Content

The maximum extraction yield of pectin from sugar beet flakes was achieved in this study using conventional citric acid extraction and was 20.80%; this result was comparable with previous reports regarding the extraction of pectin from sugar beet [30,32]. By comparison with the conventional citric acid extraction of pectin, MAE resulted in a maximum pectin yield of 7.24%, while the maximum yield achieved by PUAE in optimal extraction conditions was 3.40%. The lower pectin yield obtained by MAE and PUAE demonstrated that these non-conventional techniques did not lead to better pectin recovery, a finding that contradicts numerous studies [33,34,35]. The only improvement that was observed through the use of non-conventional extraction techniques was for the galacturonic acid content of pectin. Pectin extracted by PUAE in optimal extraction conditions had a higher GalA content (90.34 g/100 g) compared to pectin obtained using conventional citric acid extraction (76.12 g/100 g). A higher galacturonic acid content of pectin extracted by ultrasound treatment has also been reported in other studies [36].

### 2.4. The Physicochemical Properties of Pectin in Optimal Extraction Conditions

Pectin samples extracted from sugar beet flakes by CE, MAE and PUAE under the optimal conditions presented in Section 2.2 were characterized and compared with commercial pectin samples based on monosaccharide composition, degree of methylation and acetylation, molecular weight, color, thermal properties and structure (analyzed by FT-IR). The results of the analysis of these physicochemical properties are shown in Table 1.

The determination of the monosaccharide composition of pectin samples, for which the values were expressed as mol%, showed that, besides galacturonic acid, galactose, rhamnose and arabinose were the most abundant monosaccharides in pectin extracted from sugar beet flakes. Based on this, it can be assumed that pectin from sugar beet flakes consisted of homogalacturonan and rhamnogalacturonan domains with neutral side chains containing galactans and arabinogalactans, which is in line with previous reports [37]. Xylose, mannose and glucose were also detected in the samples in low concentrations. While xylose and glucose are among the neutral monosaccharides that constitute rhamnogalacturonan I [32], the presence of mannose in the samples suggested co-extraction of non-pectic polysaccharides. The main difference that was observed in the monosaccharide composition of pectin, as determined by the extraction method, was for the galacturonic acid content, which was higher in PUAE pectin. The other monosaccharides were found in different concentrations; however, the major and minor components were consistent among samples irrespective of extraction method.

The degree of methylation and acetylation of the pectin samples was determined by HPLC-RID and showed some variation in relation to the method of extraction. The highest degree of methylation and the lowest degree of acetylation were detected in pectin from CE, while the lowest degree of methylation and the highest degree of acetylation were observed in pectin extracted from sugar beet flakes using PUAE. All pectin samples extracted from sugar beet flakes were considered high-methoxyl pectins because the degree of methylation exceeded 60%. The values determined in this study for the degree of methylation and the degree of acetylation were comparable to those reported in previous studies for these parameters [30,38,39,40].

The HPSEC-RID determination of the weight-average molecular weight of pectin from sugar beet flakes highlighted that the M_w_ of pectin extracted using the non-conventional techniques MAE and PUAE was more than two times higher than the M_w_ of pectin extracted by CE and the M_w_ of commercial apple and citrus pectin. This observation regarding the M_w_ of pectin extracted from sugar beet flakes using non-conventional techniques was also made in a previous study [30]. The M_w_ values determined in this study for sugar beet pectin samples were consistent with the values reported in previous research [38,41].

As the color parameters in Table 1 show, pectin extracted from sugar beet flakes had lower values for lightness, while the commercial samples CP and AP had the highest values for this parameter. Commercial citrus pectin was lighter in color and more yellow, while commercial apple pectin had more redness. For pectin from sugar beet flakes, it was found that the color differed according to the extraction technique and most likely was influenced by the length of the extraction process. MAE, which was the shorter extraction process, produced pectin that was lighter in color and characterized by more yellowness. The same was observed for PUAE. Compared to pectin extracted using non-conventional techniques, pectin from CE was slightly darker with more red hues. These results confirmed the findings of previous studies that reported higher L* values for pectin extracted using microwave- and ultrasound-assisted extraction in comparison with pectin extracted by CE [42,43].

**Figure 4:**
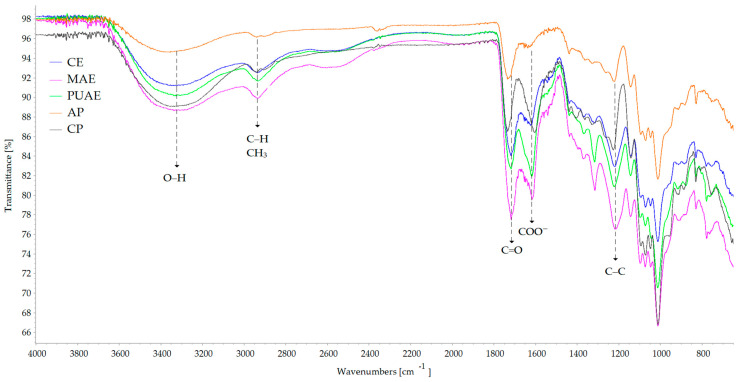
FT-IR spectra of pectin extracted from sugar beet flakes using conventional and non-conventional techniques.

FT-IR spectroscopy was used to highlight the differences determined by the extraction method in the chemical structure of pectin. The FT-IR spectra of the pectin samples presented in Figure 4 showed that the main peaks of pectin were identified in all samples regardless of the conditions of extraction.

The large absorption peak at 3320 cm^–1^ was attributed to stretching vibrations of the hydroxyl groups in the structure of pectin, and the peak at 2936 cm^–1^ was attributed to stretching vibrations of C–H involving the CH and CH_2_ groups, and also bending vibrations of CH_3_ [44,45]. The peak at 1718 cm^−1^ indicated stretching vibrations of ester carbonyl (C=O) and the peak at 1623 cm^−1^ showed carboxylate ion stretching (free carboxyl groups). The increase in the intensities and band areas of the esterified carboxyl groups at 1718 cm^−1^ was correlated with a high degree of esterification of pectin [36,46]. Furthermore, the ratio between the peak area corresponding to esterified carboxyl groups (1718 cm^−1^) and the sum of the peak areas corresponding to both free and esterified carboxyl groups (1623 cm^−1^ and 1718 cm^−1^) can be used for the quantification of the degree of esterification [47]. For pectin extracted from sugar beet flakes by CE, the peak at 1718 cm^−1^ was higher when compared with the spectra of MAE and PUAE pectin samples; this indicated a higher degree of esterification of the CE sample, which was confirmed by the analysis of the degree of methylation and acetylation. MAE and PUAE pectin samples presented a higher peak at 1310 cm^−1^ corresponding to O-acetyl-ester group stretching [48], which may be correlated with the higher degree of acetylation of these samples. All samples had peaks at 1218 cm^−1^ that were attributed to cyclic C–C bonds in the ring structure of pectin, peaks at 1072 cm^−1^ that were in the spectral range of interest for the identification of galacturonic acid, and peaks at 1012 cm^−1^ due to vibrations in the pyranose ring of pectin [49].

**Figure 5:**
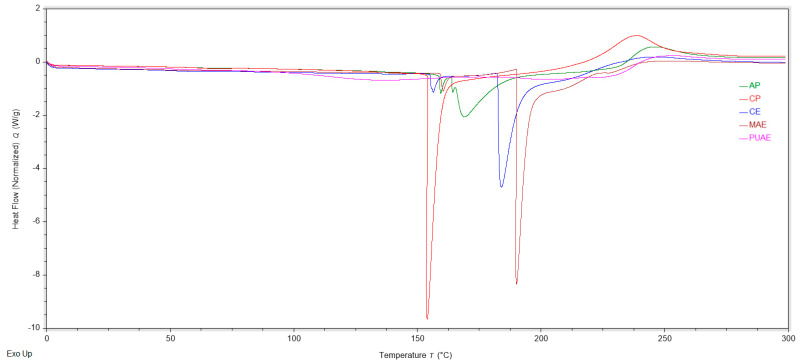
DSC thermograms of pectin extracted from sugar beet flakes using conventional and non-conventional techniques.

Thermal analysis by DSC of commercial pectin and the pectin samples extracted from sugar beet flakes by CE, MAE and PUAE, under optimal extraction conditions, was performed with the purpose of gathering information regarding the thermal behavior of the samples and to identify possible target applications for pectin extracted from this plant material. As the thermograms in Figure 5 show, in the region between 0 and 300 °C, pectin samples presented one or two endothermic peaks and one exothermic peak. For CE and MAE pectin samples, the first endothermic peak was observed at 156.42 °C and 160.45 °C, respectively, and the second endothermic peak was recorded at 183.97 °C and 190.08 °C, respectively. For PUAE pectin only one endothermic peak was observed, with a midpoint at 133.97 °C. The endothermic peaks were attributed to pectin melting and may be determined by conformational changes and the cleaving of chemical bonds that precede the degradation of the polysaccharide [50]. The thermal degradation of pectin was displayed on the thermograms as an exothermic peak at 255.12 °C for CE, 254.98 °C for MAE and 263.04 °C for PUAE. These values were higher than the thermal degradation reported for apple pectin [36], pectin from grapefruit peel [51] and pectin extracted from pomelo peel by pulsed ultrasound-assisted extraction [52], and confirm that pectin from sugar beet flakes can be used in applications that involve heat treatment.

### 2.5. Characterization of Pectin Hydrogels and Cryogels

#### 2.5.1. Rheological Properties

**Figure 6:**
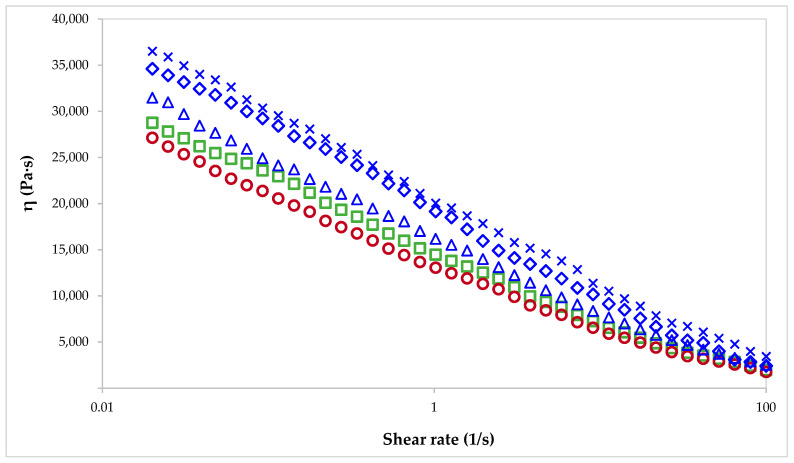
Flow curves of pectin solutions: commercial pectin AP (**○**) and CP (**☐**), and pectin extracted from sugar beet flakes by CE (**Δ**), MAE (**◇**) and PUAE (**✕**).

When pectin hydrogels and cryogels are used as components of a food product, it is important to study the rheological behavior of the pectin solution used for gel preparation, as this can influence various stages of the manufacturing process, such as mixing, pumping and storage. The rheological properties of 6% pectin solutions were characterized in this study in order to determine the differences between pectin extracted from sugar beet flakes using conventional and non-conventional methods and commercial citrus and apple pectin. The apparent viscosity of pectin solutions is presented in Figure 6. Irrespective of extraction method and pectin source, all pectin solutions showed a shear-thinning behavior that is characteristic to non-Newtonian fluids, marked by a decrease in the apparent viscosity of samples with the increase of the shear rate. The increasing shear rate caused less inter-molecular entanglement between pectin molecules, which correlated with a decrease in the viscosity of pectin solutions. Commercial pectin samples had lower viscosity than pectin extracted from sugar beet flakes. The apparent viscosity values determined for pectin extracted from sugar beet flakes were higher than those reported in previous studies for pectin extracted from sugar beet [53], mostly due to the higher pectin concentration. The extraction method determined differences in the apparent viscosity of pectin solutions, and higher values were found when pectin was obtained using non-conventional extraction methods such as microwave-assisted extraction and pulsed ultrasound-assisted extraction. When considering the physicochemical properties of pectin from sugar beet flakes, it seems that the apparent viscosity of the samples is dependent on the weight-average molecular weight, an influence also reported in other studies [36,54].

Viscosity is a parameter that also influences the applicability of pectin-based gels as 3D-printing inks. To achieve an excellent printing performance, an optimum viscosity of the pectin gel is required. High viscosities are associated with high mechanical strength, which is desired for ink deposition and self-support; however, materials with high viscosity may also clog the nozzle tip and prevent extrusion. In contrast, materials with low viscosities can be affected by the extrusion rate during 3D printing, leading to undesirable changes, such as component separation, agglomeration of solid particles, fluids release and nozzle clogging [55]. Studies published to date have reported that pectin hydrogels (containing up to 2% pectin) suitable for 3D printing had viscosity values around 10^3^ Pa∙s at low shear rates [55,56]. Because the apparent viscosity in our study exceeded this value and pectin concentration was three times higher, the 6% pectin solutions were not considered suitable for use as 3D-printing ink. However, as pectin from sugar beet flakes showed shear-thinning behavior that is greatly beneficial for the 3D-printing process, 2% pectin solutions should be considered and studied for this application.

**Figure 7:**
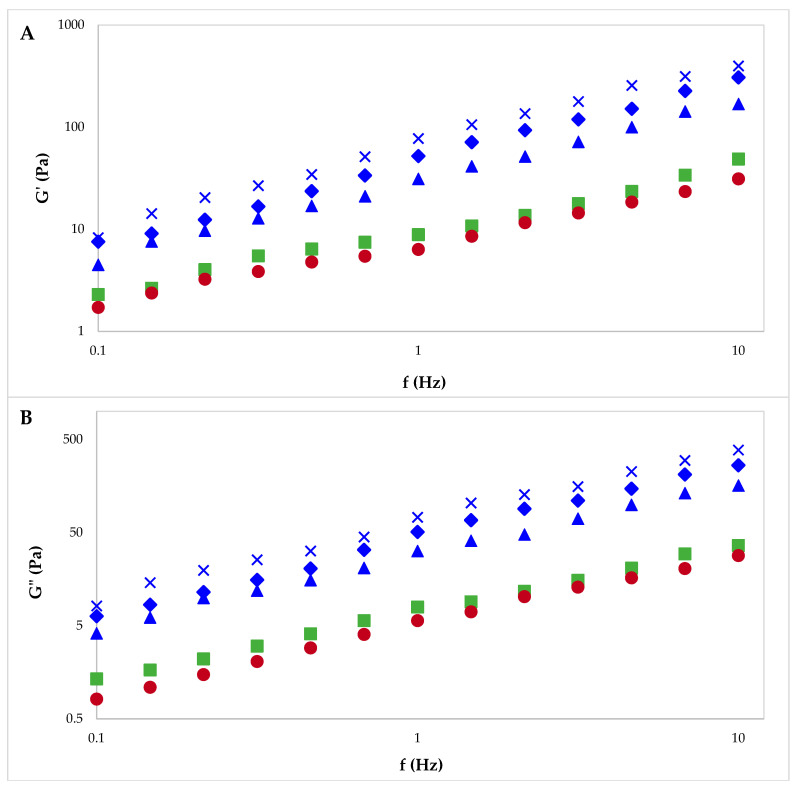
Elastic modulus (**A**) and loss modulus (**B**) for pectin solutions: commercial pectin AP (**●**) and CP (**■**), and pectin extracted from sugar beet flakes by CE (**▲**), MAE (**◆**) and PUAE (**✕**).

Frequency sweeps from 0.1 to 10 Hz (Figure 7) showed higher elastic (*G*′) and loss (*G*″) moduli for solutions of pectin from sugar beet flakes compared to commercial pectin. For all samples, *G*′ was higher than *G*″ and a gel-like behavior was observed. The viscoelastic properties of pectin extracted from sugar beet flakes using PUAE and MAE were similar, while pectin extracted by CE was more comparable to commercial citrus and apple pectin. As observed for viscosity, the *G*′ and *G*″ values seemed to vary in a similar way to the weight-average molecular weight of the samples.

#### 2.5.2. The Structural Properties of Pectin Gels

The differences in the structural properties of pectin gels as determined by the method applied for gel preparation, alongside the extraction method used to obtain pectin from sugar beet flakes are shown in Table 2. Bulk density was determined for both the “wet material”, namely the hydrogel, and the dry material, namely the cryogel, while volume shrinkage, porosity and pore volume were calculated only for the dry material. The bulk density values were similar among pectin hydrogels and cryogels; the values obtained for gels prepared with pectin from sugar beet flakes were overall slightly higher. These results were in line with the bulk density values reported for citrus pectin-based gels [57,58]. Also comparable to these studies were the volume shrinkage, porosity and pore volume determined for pectin cryogels. The limited sample shrinkage of pectin cryogels observed in this study and also reported in the literature (10–13%) was linked to the contraction of the sample upon freezing prior to sublimation in the lyophilizer [15,58]. During freezing, the growth of ice crystals in the sample causes compression on the pectin network walls and creates large pores, and as a result high porosity and high pore volume is observed for cryogels compared to aerogels and xerogels [58]. The pore structure that forms in the space occupied by ice crystals that were sublimated during freeze-drying has a great impact on the mechanical properties of cryogels [59]; higher pore volume is usually correlated with lower hardness.

As a means of increasing the mechanical strength of the gel and thus its resistance to shrinkage, the crosslinking of pectin chains can be performed through physical, chemical and interpenetrating polymer networks [15]. Crosslinking of pectin chains with divalent cations such as Ca^2+^ is the most common method and has been extensively studied; it is a process that involves mixing the pectin solution with the crosslinking ion solution (e.g., CaCl_2_), resulting in a cohesive gel structure. Gelation occurs when Ca^2+^ cations interact with the functional groups of pectin (carboxylate groups) forming scaffolds built of strong ionic bonds. Crosslinking through ionic interactions creates a 3D network that exhibits higher mechanical strength to resist shrinkage than is found in structures based on weak hydrogen bonds [59,60]. Calcium-crosslinked low-methoxyl pectin and cellulose nanocrystals hydrogels were developed as suitable food ink materials for the 3D printing of food with customizable sensory and nutritional profiles [61].

#### 2.5.3. The Color of Pectin Gels

**Figure 8:**
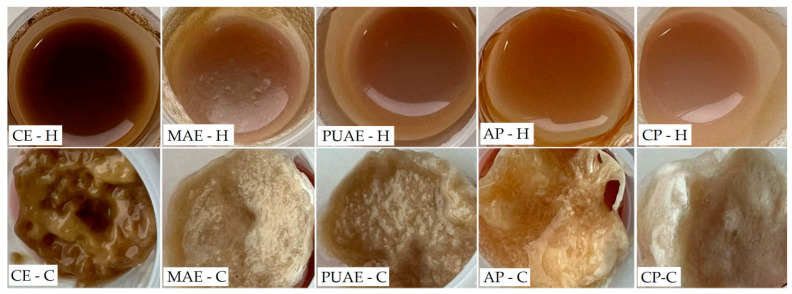
Hydrogels and cryogels prepared with pectin from sugar beet flakes and commercial pectin.

The influence of the method applied for pectin gel preparation, alongside the influence of the extraction method used to obtain pectin from sugar beet flakes, was also assessed based on the appearance of pectin hydrogels and cryogels, which was quantified through the analysis of the color parameters shown in Table 3. The hydrogels and cryogels prepared with pectin from sugar beet flakes and commercial apple and citrus pectin are shown in Figure 8. Similar to the color of pectin samples, hydrogels and cryogels obtained with commercial pectin had the highest values for lightness and were more yellow. By comparison, gels containing pectin from sugar beet flakes were darker and also characterized by more redness, and this was most notable in the case of gels made with pectin obtained using conventional extraction. Sugar beet pectin from the microwave-assisted extraction produced hydrogels and cryogels that were similar in color to gels prepared with commercial apple pectin. In terms of the differences determined by the method of gel preparation, it was observed that pectin cryogels were lighter in color and had more red and yellow hues.

#### 2.5.4. Textural Properties

A texture profile analysis using a multiple cycle compression test was performed in this study to identify the most suitable applications of hydrogels and cryogels based on pectin from sugar beet flakes in the food industry. The textural parameters of the pectin hydrogel and cryogel samples are shown in Table 4. The results obtained in this study were comparable to those reported for gels made with jackfruit waste pectin and chicory root pectin gels [62,63]. As the data in Table 4 show, hardness, which is a measure of the resistance of a pectin gel to deformation [64], varied significantly (*p* < 0.05) in relation to the method used for gel preparation and the method of pectin extraction. Cryogels showed greater hardness than hydrogels, and this may be due to the slow freeze-drying process involved in the preparation of this type of gel; this increase in the firmness and hardness of food samples as a result of slow freeze-drying has been documented by other authors [65,66]. Because gels contained only water, pectin and small amounts of citric acid, crosslinked pectin chains were strengthened and caused an increase of gel hardness. When this was combined with lower water content in the case of cryogels, it resulted in a further increase in this textural parameter, as was previously reported for composite gels [67]. Similarly, adhesiveness, stickiness, cohesiveness, gumminess and chewiness were also higher for pectin cryogels, probably also as a result of lower moisture content in these gels. Springiness was the only textural parameter that showed no significant variation between hydrogel and cryogel samples. Other authors have found springiness to be the most reproducible textural parameter compared to others such as cohesiveness and hardness [68]. Regarding the influence of the extraction method, it was observed that sugar beet pectin obtained using conventional extraction produced stronger gels, which may be due to its higher degree of methylation compared to pectin from microwave-assisted extraction and pulsed ultrasound-assisted extraction. Based on these results, gels with pectin from sugar beet flakes were considered suitable for use in the preparation of various confectionary fillings, or other applications that require the component in which pectin is added to be stable and maintain its structure following production.

#### 2.5.5. The Swelling Properties and Degradation Behavior of Pectin Gels

**Figure 9:**
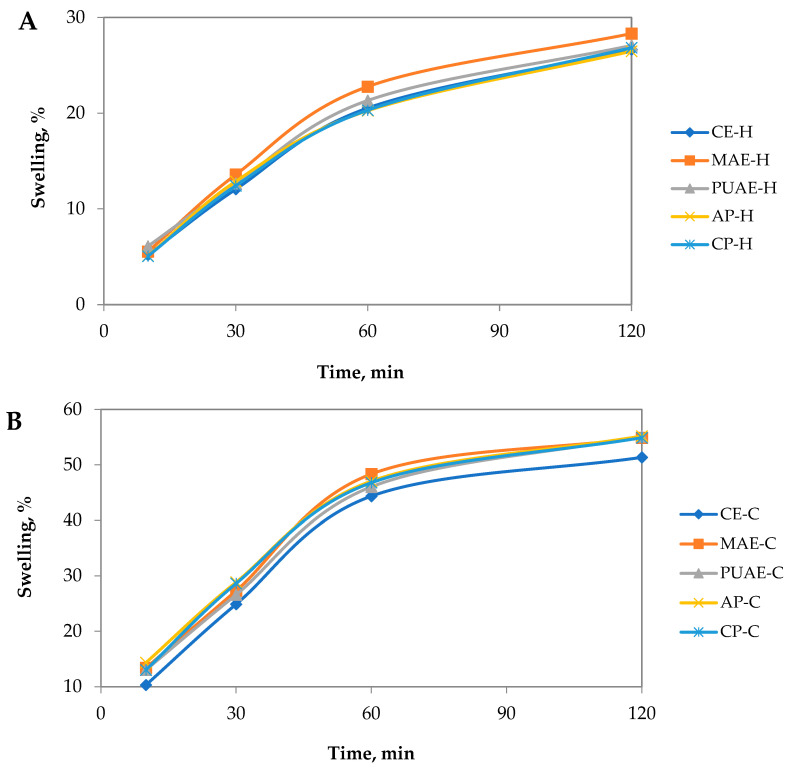
Swelling behavior of (**A**) hydrogels and (**B**) cryogels prepared with pectin from sugar beet flakes and commercial pectin.

The swelling behaviors of pectin hydrogels and cryogels were examined at 37 °C after 10 min, 30 min, 60 min and 120 min, and the results are shown in Figure 9. For all samples, a rapid swelling rate was observed in the first 60 min, with values overall higher for cryogels than hydrogels. The swelling rate of hydrogels after 2 h was between 26 and 28%, while for cryogel samples, swelling rate values after 2 h were higher and varied between 51 and 55%. For hydrogel samples, MAE-H and PUAE-H absorbed more liquid than the other hydrogels; cryogel sample MAE-C showed the highest swelling ratio, and CE-C showed the lowest swelling ratio. The different swelling ratios were attributed to the three-dimensional structure and the porosity of the pectin gels. A loosely crosslinked network with high porosity allows more liquid to be absorbed into the swollen gel [69]. The swelling ratio determined in this study for pectin gels was in line with the results reported for pectin hydrogels by other authors [70,71].

**Figure 10:**
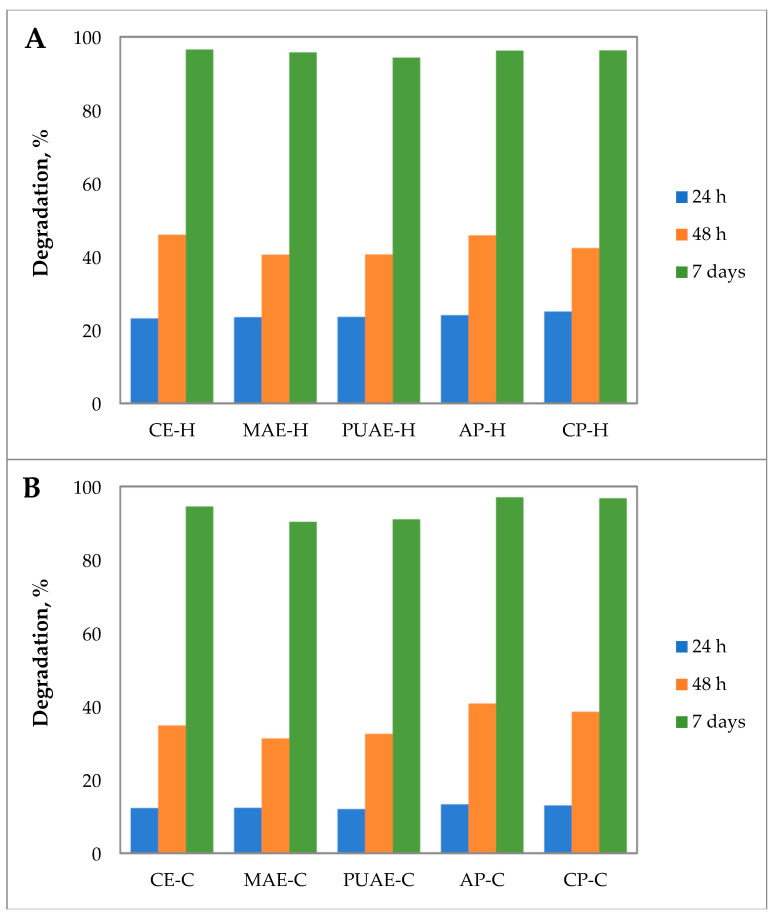
Degradation of (**A**) hydrogels and (**B**) cryogels prepared with pectin from sugar beet flakes and commercial pectin.

As shown in Figure 10, the degradation rates of gels prepared with pectin from sugar beet flakes and commercial pectin were similar and reached almost 97% on day 7 of the study. The slight differences between samples are most likely caused by the porosity and morphology of the gels. When compared to hydrogels, cryogels showed less degradation in the first 48 h; however, by day 7 the behavior was similar to that of hydrogels. Amirian et al. [72] reported degradation of 40–60% for crosslinked pectin-chitosan hydrogels on day 7, and Wang et al. [69] obtained dendrimer cryogel that completely degraded after 24 h.

#### 2.5.6. Thermogravimetric Analysis

**Figure 11:**
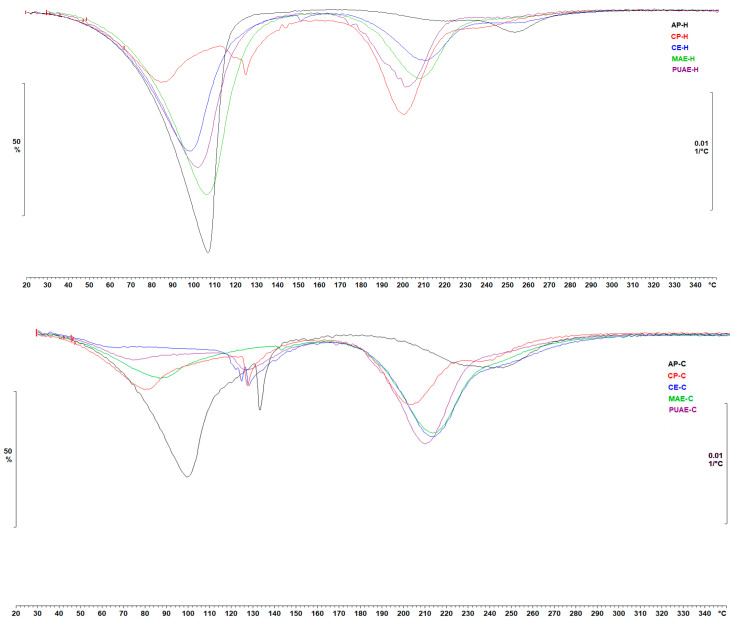
First derivative (mg/s vs. temperature) obtained from TGA of hydrogels and cryogels prepared with pectin from sugar beet flakes and commercial pectin.

The thermal analysis of pectin hydrogel and cryogel samples was performed as a means of determining their thermal stability by measuring sample weight loss versus temperature increment in the range between 30 and 400 °C. TGA thermograms (first derivative) are shown in Figure 11. All samples exhibited the first mass loss step between 41.44 and 164.07 °C, which was likely due to the removal of polymer-bound water [73]. The weight loss determined in this step for hydrogel samples (33.71–60.67%) was overall higher than the weight loss calculated for pectin cryogels (7.92–46.54%). The thermal degradation of pectin hydrogels and cryogels was indicated by the second mass loss step that occurred between 174.87 and 291.53 °C and was in line with the results of the DSC analysis performed on pectin samples; the weight loss was 9.50–27.45% for hydrogels and 15.51–35.39% for cryogels. Hydrogels and cryogels prepared with sugar beet pectin from microwave-assisted extraction and pulsed ultrasound-assisted extraction had higher weigh loss when compared to pectin extracted from sugar beet flakes using the conventional method. Furthermore, cryogels with pectin from PUAE displayed the highest thermal stability (T_max_ = 291.53 °C) of all the samples, a finding that was in line with previous studies that reported on the higher thermal stability of pectin extracted using ultrasound compared to pectin obtained using a conventional extraction procedure [51,52].

## 3. Conclusions

In this study, pectin was extracted using conventional (CE) and non-conventional extraction techniques (MAE and PUAE) from sugar beet flakes, a by-product of the sugar industry, and its gelling applications were studied through the preparation of hydrogels and cryogels. The influence of extraction conditions (temperature, pH, SLR and time for CE; microwave power, pH, SLR and time for MAE; and ultrasound amplitude, pH, SLR and time for PUAE) on pectin yield and the galacturonic acid content was confirmed to be significant. The optimization of the extraction conditions allowed further investigation into the properties of pectin. The highest extraction yield was obtained from the conventional citric acid extraction method (20.80%), and the extracted pectin had high galacturonic acid content (76.12 g/100 g) and a high degree of methylation and acetylation (70.84% and 21.90%). An improvement determined by the non-conventional methods of extraction was observed for molecular weight.

Hydrogels and cryogels prepared with pectin extracted from sugar beet flakes showed differences in physicochemical properties that were determined by both gel preparation method and method of pectin extraction. Pectin extracted using CE produced stronger gels due to the higher degree of methylation, while sugar beet pectin from non-conventional extraction methods determined solutions with higher apparent viscosity, and produced gels with higher thermal stability. Hydrogels and cryogels prepared with pectin from MAE were more comparable in appearance with gels prepared using commercial apple pectin. Hydrogels were characterized by higher bulk density values and lower hardness, adhesiveness, stickiness, cohesiveness, gumminess and chewiness than cryogels. Cryogels prepared using pectin from sugar beet flakes showed overall improved physicochemical properties (textural parameters, thermal stability) and were considered suitable for use in the preparation of various confectionary fillings or any other food component that needs to maintain its structure following production.

## 4. Materials and Methods

### 4.1. Materials

Sugar beet flakes used for the extraction of pectin were supplied by a sugar beet processing plant in the north-east region of Romania, which belongs to one of the largest sugar producers in Europe. The by-product was dried to a constant weight in a laboratory oven with air circulation ZRD-A5055 (Zhicheng Analysis Instrument, Shanghai, China) at 60 °C and then powdered; powder with particle sizes in the interval 125–200 μm was used for pectin extraction.

The reagents used in this research were of analytical grade and were purchased from Merck KGaA (Darmstadt, Germany). Commercial apple (AP) and citrus pectin (CP) and HPLC grade mobile phases used for HPLC analysis were also purchased from Merck KGaA (Darmstadt, Germany). Pullulan standards were purchased from Shodex (Tokyo, Japan). Ethyl alcohol used for pectin precipitation and purification was purchased from Redox Research & Analytic (Bucharest, Romania).

### 4.2. Procedure for Pectin Extraction and Purification

Conventional citric acid extraction (CE): for this pectin extraction procedure, sugar beet flakes powder (10 g) was mixed with water acidified with citric acid (pH of 1, 1.5 or 2) in a solid-to-liquid ratio of 1:10, 1:15 or 1:20 g/mL (*w*/*v*), and heated at temperatures of 80, 90 or 100 °C in a JP Selecta Precisdig water bath (J.P. SELECTA, Barcelona, Spain) for 60, 120 or 180 min.

Microwave-assisted extraction (MAE): similar to CE, sugar beet flakes powder (10 g) was mixed with water acidified with citric acid (pH of 1, 1.5 or 2) in a solid-to-liquid ratio of 1:10, 1:15 or 1:20 g/mL (*w*/*v*), and then exposed to microwaves in a MO17DW oven (Gorenje, Velenje, Slovenia) at power levels of 280, 420 or 560 W and exposure times of 60, 90 or 120 s. The temperature reached in the mixture at the highest microwave power level and exposure time was 120–130 °C.

Pulsed ultrasound-assisted extraction (PUAE): the extraction mixture, prepared in the same way as for the CE and MAE procedures, was heated at 60 °C and simultaneously exposed to pulsed ultrasound at amplitudes of 60, 80 or 100% for 20, 40 or 60 min. An Elma Transsonic TI-H-15 ultrasonic bath (Elma Hans Schmidbauer GmbH & Co. KG, Singen, Germany) was used for the extraction at 25 kHz and an ultrasonic power of 200 W.

After each extraction was completed, the mixtures were cooled down to room temperature (25 °C), and the liquid was separated from the plant material through centrifugation at 4000 rpm for 40 min, filtered and then mixed in a 1:1 (*v*/*v*) ratio with 96% ethyl alcohol (*v*/*v*) to precipitate pectin. The extract–alcohol mixture was refrigerated at 4–6 °C for 12 h to complete precipitation, and then pectin was separated through centrifugation (4000 rpm, 40 min), washed 3 times with concentrated ethyl alcohol for purification, and dried to a constant weight at a temperature of 50 °C in an oven with air circulation. Finally, pectin was powdered to obtain particles with sizes below 200 μm.

Pectin yield was calculated for each extraction using the equation:

(1) $$Pectin\;yield\;(\%)=\frac{m_{p}}{m}\times 100$$

where m_p_ is the weight of dried pectin (g) and m is the weight of sugar beet flakes powder used for extraction (g).

### 4.3. Analysis of the Physicochemical Properties of Pectin

The color of the pectin samples obtained from each extraction method and the color of the commercial apple and citrus pectin samples was analyzed in triplicate at 25 °C with a CR-400 chromameter (Konica Minolta, Tokyo, Japan) that was calibrated with a standard white plate. The values of CIE L*, a*, b* color coordinates, and of the parameters hue (h*_ab_) and chroma (C*_ab_) [74], were obtained from the reflection spectra of the samples with illuminant D65 and 2° observer as previously described [36].

The galacturonic acid content (GalA) of pectin was determined in triplicate using the sulfamate/*m*-hydroxydiphenyl method developed by Filisetti-Cozzi and Carpita [75]. Sample preparation was conducted as described previously [76], and the absorbance of the sample was measured with a UV-Vis-NIR spectrophotometer (Shimadzu Corporation, Kyoto, Japan).

The monosaccharide composition of pectin extracted by CE, MAE and PUAE and of commercial pectin samples was determined using the HPLC method described by Yang et al. [77], following the procedure described previously [27]. In brief, the method involves hydrolyzation of the sample into its component monosaccharides that are subsequently labelled with 1-phenyl-3-methyl-5-pyrazolone, separation of the labelled monosaccharide derivatives in a reverse-phase C18 column, and their detection via UV absorbance at 250 nm. A HPLC system (Shimadzu, Kyoto, Japan) equipped with a Zorbax Eclipse Plus C18 column (150 × 4.6 mm, 5 μm i.d.; Agilent Technologies, Santa Clara, CA, USA) and coupled on-line with a UV SPD-M-20A detector (Shimadzu, Kyoto, Japan) was used for this analysis.

The degree of methylation (DM) and acetylation (DAc) was determined simultaneously using the method proposed by Levigne et al. [78]. For this analysis, 5 mg of pectin sample (extracted using CE, MAE and PUAE, and commercial pectin) was mixed with 0.5 mL of 10 mM CuSO_4_ solution containing 25 mM isopropanol (internal standard) and 0.5 mL of 1 M NaOH that were added to achieve saponification. The mixture was allowed to react at 4 °C for 2 h, and then was centrifuged and neutralized through a LiChrolut SCX cartridge (Supelco, Sigma-Aldrich, Saint Louis, MO, USA). The neutralized supernatants were finally filtered through 0.45 μm PTFE membranes. The quantification was made with a HPLC system (Shimadzu, Kyoto, Japan) equipped with a C18 column (250 × 4.6 mm, 5 μm i.d.; Phenomenex, Torrance, CA, USA) and coupled on-line with a RID-10A refractive index detector (Shimadzu, Kyoto, Japan).

The weight-average molecular weight (M_w_) of pectin samples was determined using the high-performance size-exclusion chromatography (HPSEC) method that was previously described [36]. The HPLC system used for the analysis was equipped with a LC-20 AD liquid chromatograph, an SIL-20A auto sampler, a CTO-20AC column oven, a Yarra 3 μm SEC-2000 column (300 × 7.8 mm; Phenomenex, Torrance, CA, USA) and a KJ0-4282 SecurityGuard column protection (Phenomenex, Torrance, CA, USA), and coupled on-line with a RID-10A refractive index detector.

The structure of pectin extracted from sugar beet flakes was analyzed using Fourier transform infrared (FT-IR) spectroscopy. The spectra were recorded in transmission mode in the mid-infrared region of 4000–400 cm^−1^ at a resolution of 4 cm^−1^ with a Spectrum Two infrared spectrophotometer (Perkin Elmer, Waltham, MA, USA).

Thermal analysis of pectin was carried out using differential scanning calorimetry (DSC). Each sample was dried in an oven with air circulation, then weighted (approx. 5 mg) and hermetically sealed in an aluminum pan with a pinhole that was inserted into the DSC 25 calorimeter (TA Instruments, New Castle, DE, USA) along with an empty pan used as a reference. The samples were first equilibrated at 5 °C, and then the measurements were performed over a temperature range of 0–300 °C, at a constant heating rate of 10 °C/min using nitrogen as a purge gas at a flow rate of 20 mL/min.

### 4.4. Hydrogels and Cryogels Preparation and Analysis

Pectin hydrogels. A pectin solution of 6% (*w*/*v*) was prepared by dissolving pectin extracted from sugar beet flakes using CE, MAE and PUAE, and commercial citrus and apple pectin, in distilled water at a temperature of 65 °C and under continuous stirring (500 rpm). Then, the pH of the solution was adjusted to pH 2.0 with citric acid. This composition was poured into cylindrical molds with a diameter of 27 mm and a height of 25 mm, and kept at room temperature for 48 h to obtain hydrogels.

Pectin cryogels. Pectin hydrogels prepared as described above were at first completely frozen, and then freeze-dried in a lyophilizer model BK-FD12 (Biobase, Jinan, China) under vacuum conditions at 10 Pa and a freezing trap temperature of −50 °C for 48 h to obtain pectin cryogels.

The rheology of pectin solutions (6% pectin concentration) used to prepare hydrogels and cryogels was investigated with a Mars 40 rheometer (Thermo Haake, Burladingen, Germany) using a cone (Ø 35 mm, 2°)–plate system. The flow behavior of pectin solutions was analyzed in the controlled rate (CR) mode by ranging the shear rate (γ, s^−1^) from 0 to 100 s^−1^. Stress sweep tests of pectin solutions were performed from 0.01 to 1000 Pa at 1 Hz to determine the linear viscoelastic region; to obtain the elastic modulus (*G*′) and loss modulus (*G*″), frequency sweep tests were performed from 0.01 to 10 Hz within the linear viscoelastic region of each system [56].

The structural properties of pectin gels were determined according to the methods described by Groult et al. [58]. The bulk density of pectin gels was determined as the ratio of sample mass to sample volume. Volume shrinkage due to freeze-drying was determined using the equation:

(2) $$Volume\;shrinkage\;(\%)=\frac{V_{h}-V_{d}}{V_{h}}\times 100$$

where V_h_ is the volume of the hydrogel before drying and V_d_ is the dry network volume.

The porosity (*ε*) of pectin gels, defined as the ratio between the volume of pores and the total volume of the sample, was estimated from the bulk and skeletal densities:

(3) $$\varepsilon \;(\%)=\frac{V_{pores}}{V_{material}}\times 100=\left(1-\frac{\rho_{bulk}}{\rho_{skeletal}}\right)\times 100$$

where the skeletal density of pectin networks (ρ_skeletal_) is 1.5 g/cm^3^ [58].

The specific pore volume (V_pores_) was also estimated based on the bulk and skeletal densities:

(4) $$V_{pores}=\frac{1}{\rho_{bulk}}-\frac{1}{\rho_{skeletal}}$$

The color of the pectin hydrogels and cryogels was analyzed in triplicate using the same method as described in Section 4.3 for the color analysis of pectin samples.

The textural parameters (hardness, adhesiveness, springiness, stickiness, cohesiveness, chewiness and gumminess) of pectin hydrogels and cryogels were obtained from a multiple cycle compression analysis with a TVT 6700 texture analyzer (Perten Instruments, Hägersten, Sweden) fitted with a stainless-steel cylinder probe of 18 mm diameter. The samples were compressed up to 60% strain at a test speed of 5 mm/s at room temperature.

The swelling behavior of hydrogels and cryogels were determined according to the method described by Wang et al. [69], with some modifications. For this analysis, 0.2 g samples of hydrogels and cryogels were immersed in 1 mL of phosphate buffer saline (PBS) and incubated at 37 °C for a measurement period of up to 2 h. PBS was carefully drawn out at different time intervals (10 min, 30 min, 1 h, 2 h), and each swollen gel sample was weighed. The swelling ratio was determined using the equation:

(5) $$\mathrm{Swelling}\;\mathrm{ratio}\;(\%)=\frac{W_{t}-W_{0}}{W_{0}}\times 100$$

where W_t_ is the mass of the swollen sample and W_0_ is the mass of the dry sample.

To study the degradation of pectin gels, each sample was weighed and then saturated with PBS at 37 °C, and then 2 mL of PBS was added and incubated at 37 °C for 24 h, 48 h and 7 days, respectively. At different time intervals, PBS was carefully drawn out and samples were freeze-dried and weighed again. The degree of degradation (*D*) was calculated as:

(6) $$D\;(\%)=\frac{m_{0}-m_{de}}{m_{0}}\times 100$$

where m_0_ is the initial mass of the sample and m_de_ is the final mass of the sample.

Thermogravimetric analysis of pectin hydrogels and cryogels was performed with a TGA 2 thermal analyzer (Mettler Toledo, Columbus, OH, USA) between 30 and 400 °C at a heating rate of 10 °C/min and nitrogen flow rate of 40 mL/min.

### 4.5. Response Surface Methodology and Statistical Analysis

The extraction process was studied and optimized using a Box-Behnken response surface experimental design. For each extraction procedure, the effects of the extraction conditions (independent variables) on the extraction yield (*Y*) and the galacturonic acid content (*GalA*) of pectin (dependent variables) were studied. The independent variables were, as follows: for the CE procedure, temperature (*T*), *pH*, solid-to-liquid ratio (*SLR*) and time (*t*) (Table S1, Supplementary Materials), for MAE, microwave power (*P*), *pH*, solid-to-liquid ratio (*SLR*) and time (*t*) (Table S2, Supplementary Materials); and for PUAE, ultrasound amplitude (*A*), *pH*, solid-to-liquid ratio (*SLR*) and time (*t*) (Table S3, Supplementary Materials). Modelling and optimization were made with Stat-Ease 360 software (trial version, Stat-Ease, Minneapolis, MN, USA).

The results of the analysis of the physicochemical properties of pectin and the properties of hydrogels and cryogels were submitted to analysis of variance (ANOVA) using Statgraphics Centurion XVI software (Statgraphics Technologies, Inc., The Plains, VA, USA). Fisher’s least significant difference (LSD) procedure was used at the 95% confidence level.

## Figures and Tables

**Table 1:** Physicochemical properties of pectin extracted from sugar beet flakes and of commercial pectin samples.

	Pectin Extracted from Sugar Beet Flakes			Commercial Pectin	
	CE	MAE	PUAE	AP	CP
**Monosaccharide composition, mol%**					
Galacturonic acid	77.12 (2.24) ^d^	70.81 (1.28) ^e^	88.53 (1.05) ^a^	81.20 (1.20) ^c^	85.76 (0.84) ^b^
Arabinose	3.53 (0.02) ^d^	4.80 (0.26) ^c^	2.09 (0.13) ^e^	8.29 (0.04) ^a^	7.14 (0.11) ^b^
Galactose	7.36 (0.18) ^b^	8.84 (0.46) ^a^	4.58 (0.15) ^d^	5.21 (0.07) ^c^	0.92 (0.02) ^e^
Glucose	0.54 (0.01) ^a^	0.31 (0.00) ^c^	0.26 (0.01) ^c^	0.05 (0.00) ^d^	0.41 (0.01) ^ab^
Mannose	0.77 (0.01) ^b^	0.85 (0.01) ^a^	0.33 (0.02) ^d^	0.91 (0.02) ^a^	0.58 (0.02) ^c^
Rhamnose	5.16 (0.08) ^b^	6.67 (0.06) ^a^	2.77 (0.08) ^d^	3.70 (0.02) ^c^	0.63 (0.00) ^e^
Xylose	1.17 (0.05) ^b^	1.20 (0.03) ^b^	0.85 (0.02) ^d^	1.02 (0.01) ^c^	1.36 (0.02) ^a^
**Degree of methylation, %**	70.84 (0.10) ^b^	67.36 (0.84) ^c^	63.74 (0.62) ^c^	77.72 (0.42) ^b^	88.90 (0.30) ^a^
**Degree of acetylation, %**	21.90 (0.42) ^a^	22.29 (0.18) ^a^	22.58 (0.22) ^a^	3.14 (0.14) ^b^	1.94 (0.30) ^b^
**Molecular weight, g/mol**	1.18 × 10^5^ (0.02) ^c^	3.85 × 10^5^ (0.05) ^b^	7.40 × 10^5^ (0.07) ^a^	1.16 × 10^5^ (0.02) ^c^	1.19 × 10^5^ (0.02) ^c^
**Color parameters**					
L*	58.79 (0.96) ^d^	64.54 (0.89) ^c^	63.63 (0.25) ^c^	75.13 (0.61) ^b^	78.60 (0.08) ^a^
a*	4.16 (0.04) ^a^	2.55 (0.28) ^b^	2.97 (0.01) ^b^	4.28 (0.31) ^a^	2.42 (0.03) ^b^
b*	16.21 (0.33) ^b^	13.89 (0.14) ^c^	13.82 (0.05) ^c^	19.54 (0.20) ^a^	15.27 (0.14) ^b^
h*_ab_	75.80 (0.12) ^c^	79.59 (0.25) ^a^	77.83 (0.07) ^b^	77.64 (0.77) ^b^	80.98 (0.04) ^a^
C*_ab_	16.73 (0.43) ^b^	14.12 (0.08) ^d^	14.13 (0.04) ^d^	20.01 (0.26) ^a^	15.46 (0.14) ^c^
**ΔE**	1.44 (0.02) ^a^	1.34 (0.03) ^b^	1.39 (0.01) ^b^	1.02 (0.00) ^d^	1.23 (0.01) ^c^

^a–e^ Different letters in the same line indicate significant differences among samples (*p* < 0.05). L* represents lightness in the CIEL*a*b* system, same goes for a*, b*, h*_ab_ and C*_ab_.

**Table 2:** Structural properties of hydrogels (H) and cryogels (C) prepared with pectin from sugar beet flakes and commercial pectin. Mean values and standard deviation, in brackets.

Gel Sample	Bulk Density, g/cm^3^	Volume Shrinkage, %	Porosity, %	Pore Volume, cm^3^/g
CE-H	1.19 (0.01) ^b^	-	-	-
CE-C	0.09 (0.00) ^e^	10.80 (0.22) ^c^	93.78 (1.98) ^b^	10.05 (0.12) ^b^
MAE-H	1.22 (0.02) ^a^	-	-	-
MAE-C	0.09 (0.00) ^e^	10.67 (0.08) ^c^	93.90 (0.76) ^b^	10.27 (0.40) ^b^
PUAE-H	1.24 (0.01) ^a^	-	-	-
PUAE-C	0.09 (0.00) ^e^	10.40 (0.14) ^c^	93.82 (1.32) ^b^	10.12 (0.76) ^b^
AP-H	1.11 (0.00) ^d^	-	-	-
AP-C	0.08 (0.00) ^e^	12.00 (0.25) ^a^	94.67 (2.08) ^a^	11.83 (0.58) ^a^
CP-H	1.15 (0.01) ^c^	-	-	-
CP-C	0.08 (0.00) ^e^	11.73 (0.10) ^b^	94.58 (0.86) ^a^	11.63 (0.45) ^a^

^a–e^ Different letters in the same column indicate significant differences among samples (*p* < 0.05); H—hydrogel, C—cryogel.

**Table 3:** Color parameters of hydrogels (H) and cryogels (C) prepared with pectin from sugar beet flakes and commercial pectin. Mean values and standard deviation, in brackets.

Gel Sample	L*	a*	b*	h*_ab_	C*_ab_	ΔE
CE-H	37.13 (0.11) ^d^	6.16 (0.13) ^a^	13.39 (0.20) ^b^	65.22 (0.13) ^d^	14.74 (0.23) ^b^	0.36 (0.01) ^c^
CE-C	38.78 (0.49) ^d^	6.10 (0.07) ^a^	13.66 (0.42) ^b^	65.94 (0.40) ^d^	14.96 (0.41) ^b^	0.92 (0.07) ^a^
MAE-H	51.49 (0.19) ^b^	0.31 (0.02) ^e^	7.65 (0.33) ^d^	88.45 (1.15) ^a^	7.66 (0.33) ^d^	0.53 (0.04) ^c^
MAE-C	52.23 (0.81) ^b^	3.89 (0.01) ^c^	13.72 (0.30) ^b^	74.18 (0.34) ^c^	14.25 (0.29) ^b^	0.71 (0.06) ^b^
PUAE-H	47.62 (0.12) ^c^	0.88 (0.02) ^e^	8.39 (0.06) ^d^	84.06 (0.09) ^b^	8.44 (0.06) ^d^	0.19 (0.00) ^d^
PUAE-C	48.92 (0.40) ^c^	3.61 (0.16) ^c^	11.68 (0.28) ^c^	72.87 (0.35) ^c^	12.22 (0.31) ^c^	0.73 (0.04) ^b^
AP-H	51.49 (0.09) ^b^	2.59 (0.06) ^d^	18.76 (0.08) ^a^	82.13 (0.13) ^b^	18.94 (0.08) ^a^	0.20 (0.01) ^d^
AP-C	54.03 (0.08) ^b^	4.97 (0.01) ^b^	19.99 (0.55) ^a^	76.01 (0.31) ^c^	20.50 (0.40) ^a^	0.78 (0.05) ^b^
CP-H	53.55 (0.05) ^b^	0.09 (0.01) ^e^	6.72 (0.01) ^d^	91.02 (0.33) ^a^	6.68 (0.06) ^d^	0.07 (0.00) ^e^
CP-C	61.08 (0.10) ^a^	2.35 (0.03) ^d^	14.00 (0.29) ^b^	80.47 (0.09) ^b^	14.19 (0.29) ^b^	0.43 (0.01) ^c^

^a–e^ Different letters in the same column indicate significant differences among samples (*p* < 0.05); H—hydrogel, C—cryogel. L* represents lightness in the CIEL*a*b* system, same goes for a*, b*,h*ab and C*ab.

**Table 4:** Textural parameters of hydrogels (H) and cryogels (C) prepared with pectin from sugar beet flakes and commercial pectin. Mean values and standard deviation, in brackets.

Gel Sample	Hardness, g	Adhesiveness, J	Springiness	Stickiness, g	Cohesiveness	Gumminess, g	Chewiness, g
CE-H	78.99 (0.84) ^e^	−155.14 (1.64) ^a^	0.99 (0.01) ^a^	−51.00 (0.54) ^a^	0.31 (0.003) ^b^	277.57 (2.94) ^c^	277.02 (2.03) ^c^
CE-C	6048.06 (46.15) ^a^	−77.34 (0.82) ^d^	0.99 (0.01) ^a^	−30.97 (0.33) ^c^	0.43 (0.002) ^b^	2616.31 (27.75) ^a^	2548.69 (22.94) ^a^
MAE-H	576.93 (6.11) ^d^	−123.42 (1.31) ^b^	1.00 (0.01) ^a^	−32.00 (0.34) ^c^	0.15 (0.001) ^c^	91.58 (0.97) ^d^	90.32 (0.95) ^e^
MAE-C	1907.01 (20.22) ^b^	−102.37 (1.09) ^c^	1.00 (0.01) ^a^	−29.72 (0.32) ^c^	0.31 (0.002) ^b^	1057.16 (11.21) ^b^	1044.60 (11.07) ^b^
PUAE-H	207.31 (2.19) ^e^	−94.16 (0.99) ^d^	1.00 (0.01) ^a^	−31.00 (0.32) ^c^	0.30 (0.003) ^b^	63.90 (7.66) ^d^	63.65 (0.67) ^e^
PUAE-C	1060.00 (11.24) ^c^	−90.62 (0.96) ^d^	0.99 (0.01) ^a^	−24.00 (0.25) ^d^	0.33 (0.003) ^b^	348.57 (4.70) ^c^	348.57 (3.71) ^c^
AP-H	122.27 (1.29) ^e^	−137.04 (1.45) ^b^	0.98 (0.01) ^a^	−30.00 (0.31) ^c^	0.73 (0.007) ^a^	89.43 (0.95) ^d^	88.36 (1.09) ^e^
AP-C	585.77 (6.21) ^d^	−82.78 (0.87) ^d^	0.98 (0.01) ^a^	−21.69 (0.23) ^d^	0.79 (0.007) ^a^	174.71 (1.58) ^d^	171.27 (1.68) ^d^
CP-H	741.41 (7.86) ^d^	−129.74 (1.37) ^b^	1.00 (0.01) ^a^	−37.00 (0.39) ^b^	0.14 (0.001) ^c^	149.54 (1.16) ^d^	147.48 (4.26) ^d^
CP-C	1039.20 (11.02) ^c^	−102.71 (1.09) ^c^	0.98 (0.01) ^a^	−37.00 (0.39) ^b^	0.69 (0.007) ^a^	516.85 (4.28) ^c^	512.75 (5.44) ^c^

^a–e^ Different letters in the same column indicate significant differences among samples (*p* < 0.05); H—hydrogel, C—cryogel.

## Data Availability

All data and materials are available on request from the corresponding author. The data are not publicly available due to ongoing research using a part of the data.

## Supplementary Materials

The following supporting information can be downloaded at: https://www.mdpi.com/article/10.3390/gels10040228/s1: Table S1. Box-Behnken design with experimental results and predicted response for conventional citric acid extraction of pectin; Table S2. Box-Behnken design with experimental results and predicted response for microwave-assisted extraction of pectin; Table S3. Box-Behnken design with experimental results and predicted response for pulsed ultrasound-assisted extraction of pectin; Table S4. ANOVA for yield and galacturonic acid content of pectin from CE; Table S5. ANOVA for yield and galacturonic acid content of pectin from MAE; Table S6. ANOVA for yield and galacturonic acid content of pectin from PUAE.
