# Supplementary material for: Green Extraction of Pectin from Sugar Beet Flakes and Its Application in Hydrogels and Cryogels
Source: Gels. 2024 Mar 27;10(4):228. doi: 10.3390/gels10040228 (PMC11049022; doi:10.3390/gels10040228)
Supplement: Supplementary file 1 [file gels-10-00228-s001.zip › gels-2895734-supplementary.pdf]

gels

Supplementary Materials

Table S1. Box-Behnken design with experimental results and predicted response for conventional citric acid extraction of pectin.

Run	Independent variables				Experimental results		Predicted response	
	T, °C	pH	SLR, g/mL	t, min	Yield (%)	GalA (g/100 g)	Yield (%)	GalA (g/100 g)
1	100	1	1:15	120	28.97	29.47	30.72	26.00
2	90	1	1:15	180	26.93	71.96	26.26	67.03
3	90	1.5	1:15	120	19.67	60.43	19.67	60.43
4	90	1.5	1:10	180	10.84	82.13	8.74	86.82
5	100	1.5	1:15	180	18.07	70.26	18.98	70.68
6	90	1.5	1:10	60	8.64	50.51	9.78	46.39
7	90	1.5	1:20	180	25.78	60.62	24.62	64.60
8	90	2	1:20	120	7.52	90.62	6.72	91.25
9	90	1	1:20	120	40.10	34.11	39.11	34.39
10	90	2	1:10	120	8.19	63.62	9.33	64.20
11	80	1	1:15	120	15.79	35.31	16.31	39.89
12	90	2	1:15	180	9.22	84.14	10.63	80.12
13	80	1.5	1:15	180	7.14	53.88	8.75	53.75
14	90	1.5	1:15	120	19.67	60.43	19.67	60.43
15	100	1.5	1:10	120	9.11	49.59	8.28	52.65
16	90	1	1:10	120	12.60	58.26	13.54	58.49
17	80	1.5	1:10	120	5.73	51.76	5.43	47.32
18	90	1	1:15	60	27.11	28.80	25.57	32.10
19	80	1.5	1:20	120	8.62	50.25	9.32	46.47
20	90	1.5	1:15	120	19.67	60.43	19.67	60.43
21	80	1.5	1:15	60	5.96	45.84	5.19	46.29
22	90	1.5	1:20	60	14.78	76.39	16.86	71.56
23	90	1.5	1:15	120	19.67	60.43	19.67	60.43
24	80	2	1:15	120	3.75	46.32	1.99	49.64
25	90	1.5	1:15	120	19.67	60.43	19.67	60.43
26	90	2	1:15	60	4.07	77.37	4.61	81.58
27	100	2	1:15	120	8.98	83.55	8.45	78.82
28	100	1.5	1:20	120	27.18	52.72	27.35	56.44
29	100	1.5	1:15	60	17.29	43.66	15.83	44.66

Table S2. Box-Behnken design with experimental results and predicted response for microwave-assisted extraction of pectin.

Run	Independent variables				Experimental results		Predicted response	
	P, W	pH	SLR, g/mL	t, s	Yield (%)	GalA (g/100 g)	Yield (%)	GalA (g/100 g)
1	420	1	1:15	120	8.38	39.34	8.36	42.03
2	420	2	1:20	90	2.63	57.02	2.57	59.69
3	420	1.5	1:15	90	4.29	81.93	4.29	81.93
4	420	1.5	1:15	90	4.29	81.93	4.29	81.93
5	420	2	1:10	90	2.02	70.47	2.12	72.53
6	420	1	1:20	90	8.30	33.38	8.31	31.64
7	560	1.5	1:15	60	3.21	64.22	2.97	63.93
8	280	1.5	1:10	90	3.24	91.53	2.88	90.41
9	560	1.5	1:20	90	7.24	60.04	7.61	60.44

10	560	1	1:15	90	6.99	48.45	6.82	50.04
11	420	1.5	1:15	90	4.29	81.93	4.29	81.93
12	420	1	1:15	60	4.72	46.84	4.49	44.57
13	420	1	1:10	90	2.70	56.57	2.86	54.22
14	420	2	1:15	120	4.38	65.73	4.62	67.27
15	420	2	1:15	60	1.73	69.09	1.76	65.68
16	420	1.5	1:10	60	1.95	49.38	2.15	50.56
17	420	1.5	1:15	90	4.29	81.93	4.29	81.93
18	560	1.5	1:10	90	2.04	54.23	2.32	56.37
19	420	1.5	1:20	120	8.77	33.15	8.46	32.37
20	420	1.5	1:10	120	5.89	77.06	5.51	75.16
21	280	2	1:15	90	1.75	86.66	1.81	85.47
22	560	2	1:15	90	2.47	70.22	2.10	68.54
23	420	1.5	1:20	60	4.84	55.63	5.10	57.93
24	280	1.5	1:15	120	4.22	75.10	4.56	75.71
25	420	1.5	1:15	90	4.29	81.93	4.29	81.93
26	280	1.5	1:15	60	3.53	63.87	3.50	66.36
27	280	1	1:15	90	3.31	55.55	3.57	57.63
28	560	1.5	1:15	120	8.50	55.79	8.63	53.62
29	280	1.5	1:20	90	3.76	53.79	3.49	50.93

Table S3. Box-Behnken design with experimental results and predicted response for pulsed ultrasound-assisted extraction of pectin.

Run	Independent variables				Experimental results		Predicted response	
	A, %	pH	SLR, g/mL	t, min	Yield (%)	GalA (g/100 g)	Yield (%)	GalA (g/100 g)
1	100	1.5	1:15	60	3.48	55.83	3.45	56.32
2	80	1.5	1:15	40	3.27	91.61	3.28	91.61
3	60	1.5	1:20	40	2.85	72.42	2.92	70.54
4	80	2	1:10	40	1.73	97.44	1.81	96.95
5	60	2	1:15	40	1.85	89.33	1.79	89.65
6	100	2	1:15	40	2.22	80.71	2.24	78.94
7	100	1.5	1:15	20	1.53	62.34	1.70	60.05
8	80	1.5	1:10	20	2.13	88.83	2.08	87.06
9	80	1.5	1:15	40	3.28	91.61	3.28	91.61
10	80	1.5	1:15	40	3.28	91.61	3.28	91.61
11	60	1	1:15	40	2.67	49.83	2.70	49.98
12	60	1.5	1:10	40	2.52	70.79	2.51	70.39
13	60	1.5	1:15	20	1.98	67.16	2.06	66.68
14	80	1	1:15	60	3.25	57.05	3.18	55.84
15	80	1.5	1:15	40	3.28	91.61	3.28	91.61
16	80	1	1:15	20	2.36	40.00	2.28	40.24
17	80	1	1:20	40	3.33	40.85	3.30	41.35
18	80	2	1:15	60	2.51	79.16	2.48	80.54
19	100	1	1:15	40	2.87	40.95	2.99	39.00
20	60	1.5	1:15	60	2.47	69.09	2.36	71.39
21	80	2	1:15	20	1.37	92.33	1.33	95.16
22	80	1.5	1:10	60	2.69	69.03	2.83	65.93
23	80	1.5	1:20	60	3.66	71.61	3.77	71.76
24	80	1.5	1:20	20	2.55	48.17	2.47	49.65

25	80	1	1:10	40	3.15	55.33	3.17	57.60
26	100	1.5	1:10	40	2.80	71.98	2.62	75.48
27	100	1.5	1:20	40	3.64	41.74	3.54	43.76
28	80	2	1:20	40	2.97	83.87	3.00	81.61
29	80	1.5	1:15	40	3.28	91.61	3.28	91.61

Table S4. ANOVA for yield and galacturonic acid content of pectin from CE.

Source	Sum of squares	DF	Mean square	*F*-value	*P*-value
(A) Pectin yield, %					
Model	2246.44	14	160.46	65.15	< 0.0001
Temperature	326.67	1	326.67	132.64	< 0.0001
pH	1004.12	1	1004.12	407.72	< 0.0001
SLR	395.26	1	395.26	160.49	< 0.0001
Time	33.77	1	33.77	13.71	0.0024
Temperature × pH	15.80	1	15.80	6.42	0.0239
Temperature × SLR	57.61	1	57.61	23.39	0.0003
Temperature × time	0.04	1	0.04	0.01	0.9004
pH × SLR	198.39	1	198.39	80.55	< 0.0001
pH × time	7.10	1	7.10	2.88	0.1116
SLR × time	19.36	1	19.36	7.86	0.0141
R^2			0.9849		
$Adj\text{-}R^2$			0.9698		
$Pred\text{-}R^2$			0.9129		
(B) Galacturonic acid content, g/100 g					
Model	7344.39	14	524.60	26.90	< 0.0001
Temperature	175.49	1	175.49	9.00	0.0096
pH	2936.25	1	2936.25	150.56	< 0.0001
SLR	6.51	1	6.51	0.33	0.5725
Time	840.35	1	840.35	43.09	< 0.0001
Temperature × pH	463.76	1	463.76	23.78	0.0002
Temperature × SLR	5.38	1	5.38	0.27	0.6076
Temperature × time	86.12	1	86.12	4.42	0.0542
pH × SLR	654.08	1	654.08	33.54	< 0.0001
pH × time	331.06	1	331.06	16.98	0.001
SLR × time	561.45	1	561.45	28.79	< 0.0001
R^2			0.9642		
$Adj\text{-}R^2$			0.9283		
$Pred\text{-}R^2$			0.7935		

Table S5. ANOVA for yield and galacturonic acid content of pectin from MAE.

Source	Sum of squares	DF	Mean square	*F*-value	*P*-value
(A) Pectin yield, %					
Model	129.26	14	9.23	97.38	< 0.0001
Power	9.43	1	9.43	99.50	< 0.0001
pH	31.43	1	31.43	331.47	< 0.0001
SLR	26.11	1	26.11	275.35	< 0.0001
Time	33.87	1	33.87	357.21	< 0.0001
Power × pH	2.19	1	2.19	23.10	0.0003

Power × SLR	5.48	1	5.48	57.75	< 0.0001
Power × time	5.29	1	5.29	55.79	< 0.0001
pH × SLR	6.23	1	6.23	65.65	< 0.0001
pH × time	0.25	1	0.25	2.69	0.1233
SLR × time	0.00	1	0	0.0003	0.9873
R^2			0.9898		
Adj-R^2			0.9797		
Pred-R^2			0.9414		
(B) Galacturonic acid content, g/100 g					
Model	6957.88	14	496.99	74.20	< 0.0001
Power	450.80	1	450.80	67.31	< 0.0001
pH	1611.47	1	1611.47	240.60	< 0.0001
SLR	940.40	1	940.40	140.41	< 0.0001
Time	0.68	1	0.68	0.10	0.7544
Power × pH	21.81	1	21.81	3.26	0.0927
Power × SLR	474.15	1	474.15	70.79	< 0.0001
Power × time	96.63	1	96.63	14.43	0.002
pH × SLR	23.72	1	23.72	3.54	0.0808
pH × time	4.28	1	4.28	0.63	0.4372
SLR × time	629.01	1	629.01	93.91	< 0.0001
R^2			0.9867		
Adj-R^2			0.9734		
Pred-R^2			0.9234		

Table S6. ANOVA for yield and galacturonic acid content of pectin from PUAE.

Source	Sum of squares	DF	Mean square	*F*-value	*P*-value
(A) Pectin yield, %					
Model	11.06	14	0.79	62.76	< 0.0001
Amplitude	0.40	1	0.40	32.03	< 0.0001
pH	2.07	1	2.07	164.14	< 0.0001
SLR	1.32	1	1.32	104.84	< 0.0001
Time	3.14	1	3.14	249.51	< 0.0001
Amplitude × pH	0.007	1	0.007	0.57	0.4613
Amplitude × SLR	0.06	1	0.06	5.16	0.0393
Amplitude × time	0.53	1	0.53	42.32	< 0.0001
pH × SLR	0.28	1	0.28	22.31	0.0003
pH × time	0.01	1	0.01	1.24	0.2841
SLR × time	0.07	1	0.07	6.01	0.028
R^2			0.9843		
Adj-R^2			0.9686		
Pred-R^2			0.9097		
(B) Galacturonic acid content, g/100 g					
Model	9551.68	14	682.26	126.99	< 0.0001
Amplitude	352.84	1	352.84	65.67	< 0.0001
pH	4753.31	1	4753.31	884.73	< 0.0001
SLR	747.97	1	747.97	139.22	< 0.0001
Time	0.72	1	0.72	0.13	0.7197
Amplitude × pH	0.01	1	0.01	0.0031	0.9561
Amplitude × SLR	253.92	1	253.92	47.26	< 0.0001

Amplitude × time	17.81	1	17.81	3.31	0.0901
pH × SLR	0.20	1	0.20	0.03	0.8472
pH × time	228.31	1	228.31	42.50	< 0.0001
SLR × time	467.42	1	467.42	87.00	< 0.0001
R^2			0.9922		
Adj-R^2			0.9844		
Pred-R^2			0.9550		
